# Advancing Three-Photon-Excited Rydberg RF Sensing: Fluorescence Readout for Wide-Dynamic-Range Characterization and Spatial Resolution Beyond Transmission

**DOI:** 10.3390/s25237185

**Published:** 2025-11-25

**Authors:** Jianan Zhang, Yuqing Liu, Yimin Liu, Zhenlu Xu, Fengdong Jia, Jinghui Wang, Fei Meng, Qiang Wang, Jianwei Zhang, Zhiping Zhong

**Affiliations:** 1 School of Physical Sciences, University of Chinese Academy of Sciences, Beijing 100049, China; 2 National Institute of Metrology, Beijing 100029, China; 3 School of Mechanical and Power Engineering, Shenyang University of Chemical Technology, Shenyang 110142, China; 4 CAS Center for Excellence in Topological Quantum Computation, University of Chinese Academy of Sciences, Beijing 100190, China

**Keywords:** Rydberg atoms, fluorescence readout, microwave electrometry, wide dynamic range

## Abstract

We theoretically and experimentally investigated the fluorescence and transmission readouts of radio-frequency (RF) electrometry based on three-photon-excited Rydberg atoms. We developed a theoretical model for the fluorescence and transmission readout processes of a three-photon-excited Rydberg atom electrometer and performed a qualitative comparative analysis of fluorescence versus probe transmission readouts. Theoretical calculations revealed that while both fluorescence and probe transmission readouts can employ Autler–Townes (AT) splitting to measure strong RF fields, probe transmission readouts become ineffective in weak-field regimes, whereas fluorescence readouts remain sensitive to weak RF fields. Experimentally, we comprehensively characterize the fluorescence response across a wide range of RF field strengths: from the weak-field regime (exhibiting scaling of fluorescence peak amplitude with RF field strength), through the intermediate-field regime (where fluorescence spectral linewidth scales proportionally with RF field strength), to the strong-field regime (characterized by traditional A-T splitting). Furthermore, by adding a narrow slit in front of the photomultiplier tube (PMT) and scanning the slit together with the PMT along the light beam propagation, we exploit fluorescence’s inherent spatial information to directly map the Rydberg excitation profile and local RF field strength. This overcomes the transmission readout’s inherent limitation of providing only path-integrated signals along the probe beam, even by imaging the probe beam with a CCD camera. Our results establish fluorescence readouts as a superior technique for three-photon Rydberg electrometry, offering enhanced wide-range RF field sensing and direct spatial field mapping.

## 1. Introduction

Quantum precision measurements utilize quantum resources and effects to achieve high measurement accuracy beyond classical limits, with the goal of discovering new physical laws [1], testing fundamental physical theorems [2], measuring and calibrating fundamental physical constants [3], and refining the International System of Units (SI) [4]. In particular, quantum sensors have achieved rapid development recently and have obtained outstanding achievements in fields such as time and frequency measurement [5,6,7], gravity measurement [8,9,10,11], magnetic-field measurement [12,13,14], and electric-field measurement [15,16,17]. Radio-frequency (RF) electric fields, defined as electromagnetic waves spanning 300 MHz to 300 GHz, are of critical importance across diverse fields, including electronic information, aviation, aerospace, biomedical science, and so on. In 2012, Sedlacek et al. demonstrated absolute traceable measurement of RF field strength using Rydberg-atom-based electromagnetically induced transparency (EIT) in a rubidium (Rb) vapor cell, where the field strength was linear, with the Autler–Townes (AT) splitting interval induced by RF fields [15]. This landmark work has since stimulated extensive research and significant progress in the RF electrometry field. When the RF field strength is weak, the conventional EIT-AT splitting method fails due to its inability to produce a distinguishable splitting interval [18]. To overcome this limitation, researchers have developed various enhanced techniques for weak-field measurements, including frequency-detuned RF technique [19,20], magnetic-field-modulated detection [21,22,23,24], auxiliary-field expansion [25,26,27,28,29], microwave amplitude modulation [30,31], polarization spectroscopy [32], and cold-atom-based methods [33,34,35]. While these methods extended the lower detection limit of RF field measurement via EIT-AT splitting, they eventually fail as the field strength decreases further, driving researchers to explore new measurement strategies when EIT-AT splitting becomes completely ineffective. For instance, the changes of the transmission of probe light at the resonance point are used to detect RF fields, such as variations in the transmission of the probe light at resonance [15] and frequency-modulated dispersion spectroscopy [36]. A distinguished work in addressing weak-field measurements is the atomic superheterodyne method using a local RF field, proposed by Jing et al. in 2020 [16]. This method successfully amplifies the weak signals of RF fields, enabling precise measurements at extremely weak field strengths. Almost simultaneously, Gordon et al. also demonstrated an experiment using an atomic mixer to measure weak RF fields [37]. In recent years, researchers have proposed various schemes to improve superheterodyne sensitivity, including microwave amplitude modulation [38], microwave cavity enhancement [39,40], probe optical cavity enhancement [41], and interferometer readouts [42]. In addition, Ding et al. use multi-body interaction critical points to enhance the sensitivity of Rydberg-atom-based electrometry [17].

Rydberg-atom-based electrometry typically employs two-photon (infrared + visible) or all-infrared three-photon excitation EIT schemes. The fundamental configuration involves a collinear arrangement of probe and coupling laser beams that co-propagate through an atomic vapor cell, where they interact with atoms and the RF field. The RF field strength is detected by measuring the resultant variations in the transmission intensity of the probe beam using a photodetector, that is, via probe light transmission readout. This non-contact optical reading scheme has the advantages of laser directionality and convenient measurement, allowing the RF field strength to be measured simply by directly detecting the probe light intensity with a photodetector. Otherwise, accurately controllable laser beams enable high-spatial-resolution measurement at sub-wavelength microwave scales as well [43,44]. But probe light transmission readouts also have inherent limitations: (1) when detecting probe light transmission after a vapor cell, only a very small fraction of photons (less than 1%) participate in the interaction, resulting in an EIT signal with a large background and, consequently, low contrast; (2) the spatial resolution is inherently limited to the direction perpendicular to the laser beam, meaning that the measurement represents an integrated signal along the entire light path [43], which can only resolve the RF field distribution perpendicular to the propagation direction by imaging the probe beam with a CCD camera [44].

Atoms in excited states can undergo transitions to lower energy states through spontaneous emission, thereby emitting fluorescence. The properties of the excited states can be detected by this fluorescence. The Laser-Induced Fluorescence (LIF) technique offers high temporal and spatial resolutions, and has been widely successful in fields such as plasma diagnostics and quantum gas preparation [45]. It has achieved single-quantum-state resolution (with a temporal resolution < 1 μs) and sub-micrometer spatial localization. In practice, LIF detection often employs fluorescence signals at wavelengths different from those of excitation lasers to suppress background noise such as scattered light. In 2024, Prajapati et al. proposed a fluorescence readout method based on an all-infrared three-photon excitation scheme [46], which detects visible fluorescence and effectively avoids noise caused by the wavelength overlap of fluorescence and excitation lights. Their outstanding work demonstrated a direct correlation between the fluorescence intensity and the strength of the RF field. Compared to conventional three-photon EIT and electromagnetically induced absorption (EIA) techniques, their method achieves nearly a fourfold improvement in sensitivity, offering a more practical technological scheme for high-sensitivity and low-noise electric field detection. As far as we know, no article has provided a theoretical model of this fluorescence-readout scheme, nor sufficiently established its suitability for wide dynamic range characterization. Furthermore, the potential of fluorescence readouts for spatially resolved RF field detection remains unexplored.

This work can be regarded as further research of the reference [46]; we conducted a further investigation into a comparative scheme between fluorescence and probe transmission detection of a Rydberg-atom-based electrometry to demonstrate the key advantages of fluorescence-based measurements. The structure of the study is as follows: Section 2 starts with theoretical models, and explains the response differences between fluorescence and probe transmission detection in a wide-dynamic-range RF field. Section 3 introduces the experiment setup and measurement process. Section 4 compares the RF field measurement results between fluorescence and probe transmission detection, and represents a preliminary attempt to measure the RF field spatial resolution along the direction of laser transmission by fluorescence detection. Section 5 summarizes the conclusions.

## 2. Theoretical Model

**Figure 1:**
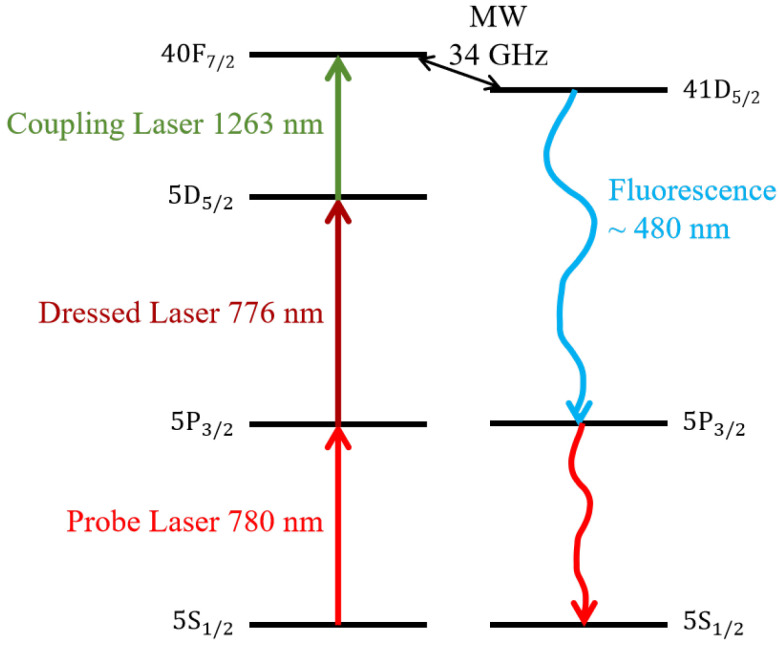
Rydberg atomic excitation and fluorescence channels.

This work is based on theoretical and experimental research on atoms in a Rb vapor cell at room temperature. Figure 1 shows the relevant five energy levels and channels for excitation and fluorescence radiation. Specifically, atoms start from the ground state $5S_{1/2}$ and are sequentially excited by probe light (780 nm), dressed light (776 nm), and coupling light (1263 nm) to $5P_{3/2}$, $5D_{5/2}$, and the Rydberg state $40F_{7/2}$, and then coupled to $41D_{5/2}$ by a microwave (MW) field within the RF band at 34 GHz. The lasers and microwave are vertically polarized, permitting the treatment of the system as a five-level model [15,47]. Fluorescence radiation involves three levels, of which atoms at $41D_{5/2}$ transition to $5P_{3/2}$ by spontaneous emission and emit fluorescence with a wavelength of about 480 nm. This fluorescence has a different wavelength from the excitation light for the convenience of light filtering during fluorescence detection.

In 2024, Miller et al. released an open-source Python library named RydIQule (version 2.1.0), designed for computing the response of Rydberg-based sensors to arbitrary MW fields [48]. RydIQule is a density matrix-based master equation solver designed to rapidly solve problems with a large parameter space while maintaining the flexibility to accommodate user-defined scenarios and novel challenges. We use RydIQule to build an excitation model, as shown in Figure 1, and we calculate the relevant density matrix elements starting from the master equation [47]:

(1) $$\dot{\rho }=\frac{\partial \rho }{\partial t}=-\frac{i}{\hbar }\left[H,\rho \right]+L,$$

where *H* denotes the system’s Hamiltonian and L denotes the Liouvillian superoperator in Lindblad form, which accounts for dissipative processes (atomic decay). For the 5-level system, the Hamiltonian is as shown:

(2) $$\;H=\hbar \;\left[\begin{array}{ccccc} 0 & \frac{\Omega_{p}}{2} & 0 & 0 & 0\\ \frac{\Omega_{p}}{2} & -\Delta_{p} & \frac{\Omega_{d}}{2} & 0 & 0\\ 0 & \frac{\Omega_{d}}{2} & -(\Delta_{p}+\Delta_{d}) & \frac{\Omega_{c}}{2} & 0\\ 0 & 0 & \frac{\Omega_{c}}{2} & -(\Delta_{p}+\Delta_{d}+\Delta_{c}) & \frac{\Omega_{\mathrm{MW}}}{2}\\ 0 & 0 & 0 & \frac{\Omega_{\mathrm{MW}}}{2} & -(\Delta_{p}+\Delta_{d}+\Delta_{c}+\Delta_{\mathrm{MW}}) \end{array}\right].$$

Here, $\Omega_{p}$, $\Omega_{d}$, $\Omega_{c}$, $\Omega_{\mathrm{MW}}$ represent the rabi frequency of probe (c), dressed (d), coupling (c), and MW, respectively. $\Delta_{p}$, $\Delta_{d}$, $\Delta_{c}$, $\Delta_{\mathrm{MW}}$ correspond to the detuning of each light. The level structure is $|1\rangle \overset{\Omega_{p}}{\to }|2\rangle \overset{\Omega_{d}}{\to }|3\rangle \overset{\Omega_{c}}{\to }|4\rangle \overset{\Omega_{\mathrm{MW}}}{\to }|5\rangle$. The diagonal elements of the matrix represent the energy detuning of each energy level, while the non-diagonal elements represent the coupling strength between energy levels. The detuning of each field is defined as:

(3) $$\begin{array}{cc} \Delta_{p} & =\omega_{p}-\omega_{0_{p}},\\ \Delta_{d} & =\omega_{d}-\omega_{0_{d}},\\ \Delta_{c} & =\omega_{c}-\omega_{0_{c}},\\ \Delta_{\mathrm{MW}} & =\omega_{\mathrm{MW}}-\omega_{0_{\mathrm{MW}}}, \end{array}$$

where $\omega_{0_{p}}$, $\omega_{0_{d}}$, $\omega_{0_{c}}$, and $\omega_{0_{\mathrm{MW}}}$ correspond to the resonance transition frequency of each light. For the five-level system, the L matrix is given by:

(4) $$L=\left[\begin{array}{ccccc} \Gamma_{2}\rho_{22} & -\gamma_{12}\rho_{12} & -\gamma_{13}\rho_{13} & -\gamma_{14}\rho_{14} & -\gamma_{15}\rho_{15}\\ -\gamma_{21}\rho_{21} & \Gamma_{3}\rho_{33}-\Gamma_{2}\rho_{22} & -\gamma_{23}\rho_{23} & -\gamma_{24}\rho_{24} & -\gamma_{25}\rho_{25}\\ -\gamma_{31}\rho_{31} & -\gamma_{32}\rho_{32} & \Gamma_{4}\rho_{44}-\Gamma_{3}\rho_{33} & -\gamma_{34}\rho_{34} & -\gamma_{35}\rho_{35}\\ -\gamma_{41}\rho_{41} & -\gamma_{42}\rho_{42} & -\gamma_{43}\rho_{43} & \Gamma_{5}\rho_{55}-\Gamma_{4}\rho_{44} & -\gamma_{45}\rho_{45}\\ -\gamma_{51}\rho_{51} & -\gamma_{52}\rho_{52} & -\gamma_{53}\rho_{53} & -\gamma_{54}\rho_{54} & -\Gamma_{5}\rho_{55} \end{array}\right].$$

Here, the coefficient $\gamma_{ij}$ is given by the mean of the decay rates $\Gamma_{i}$ for the respective transitions, that is, $\gamma_{ij}=\left(\Gamma_{i}+\Gamma_{j}\right)/2$. In order to examine the intrinsic constraints of Rydberg–EIT-based electric field sensing in vapor cells, the model intentionally neglects collisional and dephasing effects, which is often reasonable under the weak-field approximation. Radiative relaxation is taken into account, assuming that the model of 5-level atoms is closed. This is justified by the large lifetime difference between the low-lying states (10–100 ns) and the long-lived Rydberg levels (tens of μs), which makes population leakage negligible for the spectral behavior of interest. In the calculation RydIQule can call the parameters of atomic energy levels and radiative decay rates provided by the ARC program [49], which are not described in detail here.

The probe light transmission can be calculated by $\rho_{21}$:

(5) P=P0exp−Lλp·4πN0ϵ12ϵ0Ep·Im(ρ21D),

(6) $$N_{0}=\frac{0.7217\cdot 10^{5.006+4.857-\frac{4215}{T}}}{k_{B}T}.$$

Here, $P_{0}$ is the probe light incident power. *L* is the length of the vapor cell, set to 5 cm in the program. $\lambda_{p}$ is the probe light wavelength at 780 nm. $\epsilon_{12}$ is the transition dipole moment of |1〉−|2〉. $E_{p}$ is the amplitude of the probe light field. $\epsilon_{0}$ is the vacuum dielectric constant. $\rho_{21_{D}}$ is the non-diagonal elements of the density matrix after Doppler broadening, which can use the ones after Doppler convolution in RydIQUE. $k_{B}$ is the Boltzmann constant. *T* is the absolute temperature (K), taken as 300 K in the calculation. To minimize Doppler broadening, the coupling light (1263 nm) co-propagates with the dressed light (776 nm) and counter-propagates relative to the probe (780 nm).

**Figure 2:**
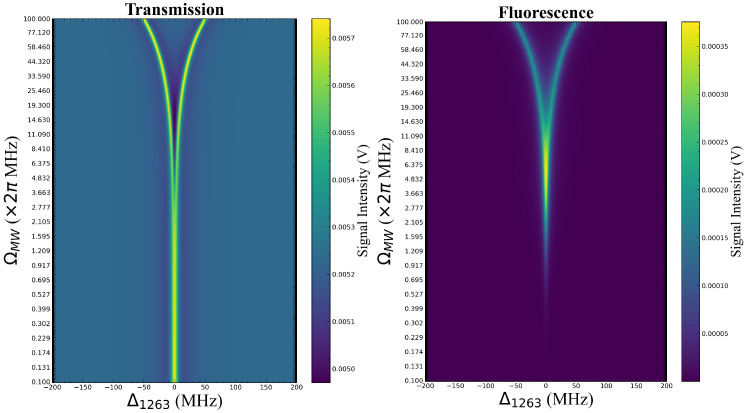
Artificial color map of a comparison of the theoretical results between transmission and fluorescence spectra. The transmission spectrum is shown on the left, and the fluorescence spectrum is shown on the right. The x-axis shows the detuning of the coupling light, the y-axis shows the variation in the MW fields, and the color intensity corresponds to the EIT signal intensity. The Y-axis is on a logarithmic scale. The main parameters are set as follows: $\Omega_{p}=2\times 2\pi$ MHz, $\Omega_{d}=2\times 2\pi$ MHz, $\Omega_{c}=5\times 2\pi$ MHz, $\Delta_{p}=0$, $\Delta_{d}=0$, and $\Delta_{\mathrm{MW}}=0$. MW Rabi frequencies $\Omega_{\mathrm{MW}}$ are set from 0.1 to 100×2π MHz in logarithmic intervals.

Fluorescence can be calculated by $\rho_{55}$:

(7) $$I_{\mathrm{fluor}}=\rho_{55}\cdot \Gamma_{52},$$

where $I_{\mathrm{fluor}}$, $\rho_{55}$, and $\Gamma_{52}$ represent the fluorescence intensity, population of the Rydberg state |5〉, and spontaneous emission rate from |5〉 to |2〉, respectively. In calculation, we set $\Omega_{p}=2\times 2\pi$ MHz, $\Omega_{d}=2\times 2\pi$ MHz, $\Omega_{c}=5\times 2\pi$ MHz, $\Delta_{p}=0$, $\Delta_{d}=0$, and $\Delta_{\mathrm{MW}}=0$. Then by changing $\Delta_{c}$ and obtaining the variation in $\rho_{21_{D}}$ and $\rho_{55}$, the information on the probe light transmission and fluorescence spectra after interaction with MW can be acquired. The MW Rabi frequencies $\Omega_{\mathrm{MW}}$ are set from 0.1 to 100 ×2π MHz in logarithmic intervals during calculation, and the probe light transmission and fluorescence spectra can be obtained by Formulas (5) and (6). To clearly demonstrate the evolution of the transmission and fluorescence spectra under different $\Omega_{\mathrm{MW}}$, we combined the calculation results at different $\Omega_{\mathrm{MW}}$ into the artificial color map, as shown in Figure 2. From the qualitative results, it can be seen that when under the weak field ($\Omega_{\mathrm{MW}}<2.2\times 2\pi$ MHz), the probe light transmission does not change significantly as $\Omega_{\mathrm{MW}}$ increases, while fluorescence intensity increases, which shows the greater advantages of the fluorescence spectrum in weak-field detection. When under strong-field conditions ($\Omega_{\mathrm{MW}}>2.2\times 2\pi$ MHz), probe light transmission presents typical EIT-AT splitting, while fluorescence spectrum is split at a higher MW field ($\Omega_{\mathrm{MW}}>8.8\times 2\pi$ MHz). Because factors such as laser noise, collisional and dephasing effects, spatial distribution of MW fields, and the solid angle of fluorescence collection in actual experiments are not considered in the theoretical model, the theoretical results in Figure 2 only provide qualitative conclusions, but still have enough guiding significance for the experimental setup.

## 3. Experimental Setup

**Figure 3:**
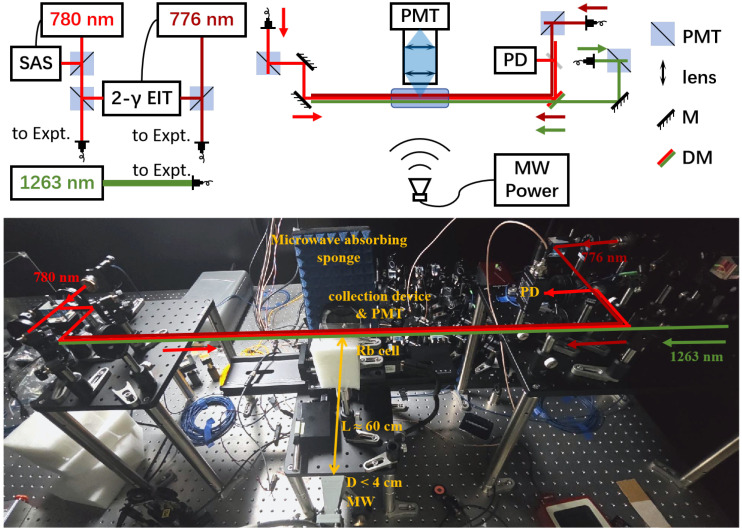
Experimental setup for simultaneously measuring the probe light transmission and fluorescence. A schematic diagram is shown in the **upper** panel, and a photograph of the setup is shown in the **lower** panel.

The experiment setup is shown in Figure 3, and the energy levels are shown in Figure 1. The four steps of excitation are as follows: $|5S_{1/2}\rangle \overset{\mathrm{probe}}{\to }|5P_{3/2}\rangle \overset{\mathrm{dressed}}{\to }|5D_{5/2}\rangle \overset{\mathrm{coupling}}{\to }|40F_{7/2}\rangle \overset{\mathrm{MW}}{\to }|41D_{5/2}\rangle$. The probe light and the dressed light are overlapped in a counter-propagating configuration within the vapor cell, while the coupling light and the dressed light are combined via a dichroic mirror and co-propagate collinearly in the same direction. MWs are incident through a horn antenna perpendicular to the direction of laser propagation, with the vertical linear polarization direction consistent with the three light beams. The probe light frequency is locked at the transition $|5S_{1/2}\rangle \to |5P_{3/2}\rangle$ by the saturated absorption spectrum, the dressed light frequency is locked at the transition $|5P_{3/2}\rangle \to |5D_{5/2}\rangle$ by 780–776 two-photon EIT, and the MW source frequency is calibrated through EIT-AT splitting symmetry [50]. The coupling light frequency is scanned by changing the voltage of the piezoelectric (PZT) for the 1263 nm laser. The system parameters are as follows: the power of the probe light is 70 μW with a diameter of 1.0 mm, the power of the dressed light is 7 mW with a diameter of 1.0 mm, and the power of the coupling light is 50 mW with a diameter of 1.0 mm. The lasers have typical linewidths of less than 1 MHz. An MW source is used to drive a horn antenna at 34 GHz. The horn antenna has an aperture diameter of 4 cm. A cylindrical Rb cell with a length of 50 mm and a diameter of 25 mm is set over 60 cm away from the MW antenna to satisfy the far-field condition. We use a foam mount to hold the Rb cell, and place a microwave-absorbing sponge behind the cell to dampen reflected waves.

In the probe light transmission scheme, we spatially separated the probe light and the dressed light using a slide glass positioned at a 45° angle to the light path, reflecting a portion of the probe light into the photodetector (PDA36A2, Thorlabs, Newton, NJ, USA) to obtain the probe light transmission spectra. In order to achieve fluorescence detection at the same time as the probe light transmission readout, we set up a coaxial fluorescence collection system in conjunction with a photomultiplier tube (PMT, PMM01, Thorlabs, Newton, NJ, USA) to obtain the fluorescence spectrum. The coaxial system and PMT are installed together on a sliding rail, which can move along the direction of laser propagation. We install two lenses with a diameter of 50 mm and a focal length of 50 mm in a coaxial sleeve to collect the fluorescence and to shine it onto the photosensitive surface of the PMT. A filter with a 10 nm bandwidth, centered at 480 nm, is used in front of the PMT to reduce stray background lights. The PMT operating voltage is set at 1.3 kV to ensure that the fluorescence signal remains in the linear gain range.

In the experiment, we will first investigate the performance of transmission and fluorescence spectra under different MW field strengths. We change the output power of the MW source from −135 dBm to 18 dBm, and record the transmission and fluorescence spectra under different MW fields by scanning the coupling light frequency. The coupling light frequency is calibrated by fluorescence spectra, which will be introduced in Section 4. In order to study the spatial resolution of fluorescence detection, we installed a 5 mm wide slit in front of the fluorescence collection system in close proximity to the vapor cell, perpendicular to the direction of laser propagation, and used a slide rail to detect the spectra at different positions along the laser propagation. During this process, the MW source power is maintained at −25 dBm to ensure that the fluorescence peak amplitude remains unsaturated. In this experiment, a longer cylindrical Rb cell with a length of 75 mm is used.

## 4. Results and Discussion

**Figure 4:**
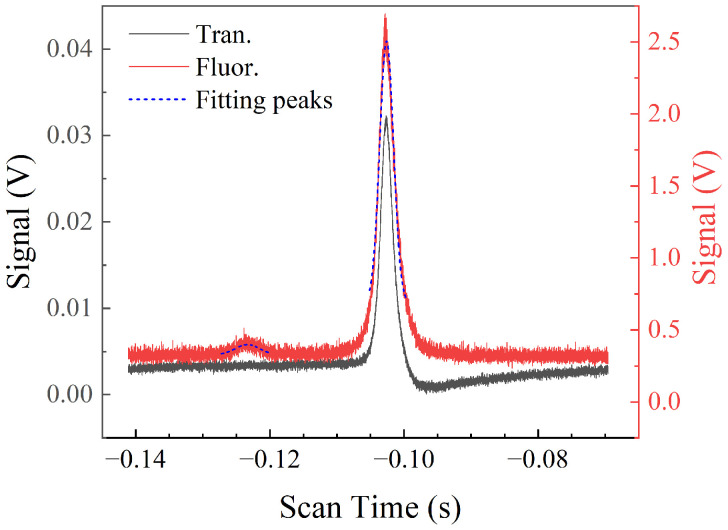
Comparison of experimental results between typical transmission and fluorescence spectra. The black line describes the transmission spectrum, the red line describes the fluorescence spectrum, and the blue dashed line describes the fitted peaks of the fluorescence spectrum.

We first calibrate the scan frequency of the coupling light by the fluorescence spectra structure. Usually, several techniques are commonly employed to calibrate unknown laser frequencies by referencing them to known standards, including acousto-optic modulator (AOM) [51] or electro-optic modulator (EOM) frequency modulation [52], optical cavity methods [53], optical frequency combs [54], reference laser beat-note techniques [55], wavemeter-based measurements [56], and so on. Figure 4 shows the simultaneously measured transmission and fluorescence spectra under an MW power output set at −35 dBm. The horizontal axis represents the scanning time of the coupling light PZT, and the vertical axis represents the signal intensity. The black line describes the transmission spectrum, and it can be seen that there is only a single EIT peak. The red line describes the fluorescence spectrum, and it is surprising that there are two peaks fitted by the blue dashed line. Analyzing the cause of two peaks can be used to calibrate the frequency of the coupling light, which represents a unique advantage inherent to fluorescence spectroscopy. The analyzed cause of of two peaks is as follows: the higher peak corresponds to the transition $|5D_{5/2}\rangle \to |40F_{7/2}\rangle \to |41D_{5/2}\rangle ⇝|5P_{3/2}\rangle$, while the lower peak corresponds to the transition $|5D_{5/2}\rangle \to |40F_{5/2}\rangle \to |41D_{3/2}\rangle ⇝|5P_{3/2}\rangle$. The two peaks are separated by 173.58 MHz with ARC calculation [57], which achieves calibration of the coupling light frequency. Due to the low transition probability of $|5D_{5/2}\rangle \to |40F_{5/2}\rangle \to |41D_{3/2}\rangle$, this peak is obscured by a strong background signal and cannot be clearly distinguished in the probe light transmission spectra. Fluorescence spectra fully utilize the single-photon detection capability of PMTs; its high sensitivity to weak signals allows low-intensity transition signals to be enhanced, resulting in a distinct two-peak structure suitable for frequency calibration. This displays its unique advantage in the field of spectral characterization.

**Figure 5:**
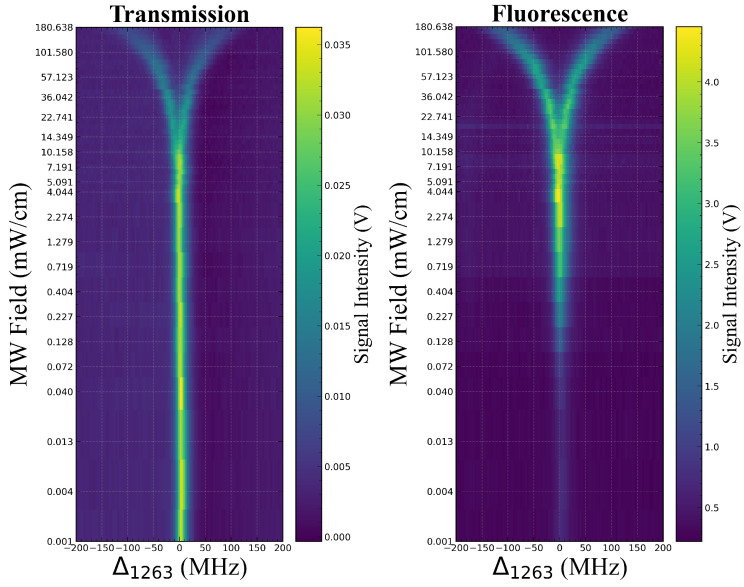
Artificial color map of comparison of the experimental results between transmission and fluorescence spectra under different MW field strengths. The transmission spectrum is shown on the **left**, and the fluorescence spectrum is shown on the **right**. The x-axis shows the detuning of the coupling light, the y-axis shows the variation in the MW fields, and the color intensity corresponds to the EIT signal intensity. The Y-axis is on a logarithmic scale.

For different MW field powers, we scan the coupling light frequency and simultaneously record the signals of the transmission and fluorescence spectra. Each set of spectra is averaged through 32 measurements to improve the signal-to-noise ratio. Subsequently, the spectra measured at different MW field powers were combined into an artificial color map (Figure 5) to visually display the dynamic evolution process of the transmission spectrum and fluorescence spectrum as the MW field power changed. Figure 5 clearly shows the evolution of both the transmission and fluorescence spectra with the MW field strength, calibrated by EIT-AT splitting of the fluorescence spectra. The experimental results exhibit the same trend as the theoretical predictions. The transmission spectrum exhibits EIT peaks that are essentially constant under weak MW conditions and broaden until splitting as the MW field strength gradually increases. The fluorescence spectrum first exhibits a process of increasing intensity and then broadens until splitting. The behavior of the transmission and fluorescence spectra under strong MW fields is similar, both showing broadening and splitting caused by the AT effect. However, in weak MW fields, the fluorescence spectrum can directly detect the population of Rydberg states and reflect more MW field strength information from the peak amplitude. More detailed descriptions of spectral characteristics will be provided, such as peak amplitude, linewidth, and AT splitting interval.

**Figure 6:**
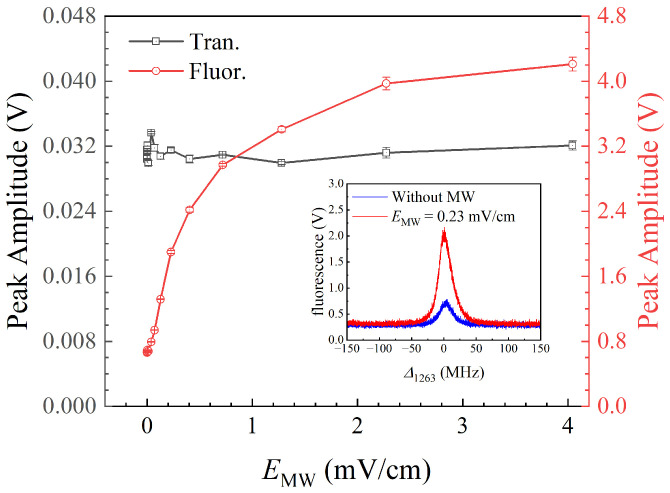
The peak amplitude variation in the transmission and fluorescence spectra under weak MW fields from 0 to 4.0 mV/cm. The black hollow square represents the transmission readout, the red hollow circle represents the fluorescence readout, and the error bar is given by the short line. The inset shows the fluorescence spectra with an MW field strength of 0.23 mV/cm (red line) and without the MW field (blue line).

Figure 6 shows experimental results of the peak amplitude variation of fluorescence and transmission spectra with MW field strengths from 0 to 4.0 mV/cm (corresponding to a MW source power set from −135 to −15 dBm). The black hollow square represents the transmission readout, the red hollow circle represents the fluorescence readout, and the error bar is given by the short line. In this range, as the MW field strength increases, the peak amplitude of the transmission spectra remains constant, while that of the fluorescence spectra increases and reaches saturation after 4.0 mV/cm, and then, it decreases due to the splitting of Rydberg energy levels, which is consistent with the trend of the transmission spectrum. Under a weak MW field (<4.0 mV/cm), the increase in the MW field strength enhances the coupling between Rydberg energy levels, thereby elevating the population of the upper energy levels and increasing the fluorescence intensity. Therefore, in this range, the peak amplitude of the fluorescence spectral peak can be used to measure MW fields. Significantly, the fluorescence peak amplitude remains almost unchanged under the MW field, ranging from 0 to 0.04 mV/cm. As shown in the inset in Figure 6, even when the MW source is turned off, a residual spectral peak can still be observed. This weak signal is attributed to processes such as blackbody radiation (BBR)-induced excitation and interatomic collisions [46], which transfer a small population of atoms from the 40F to the 41D Rydberg state, emitting fluorescence by spontaneous emission. According to the ARC calculator, the spontaneous and induced transition rates are 5.93 $s^{-1}$ at 0 K and 1082.15 $s^{-1}$ at 300 K [57]. This confirms that the population loss (and, thus, the observed fluorescence) for 40F is dominated by BBR-driven transitions to 41D, rather than by spontaneous emission back to the lower state. This result demonstrates that fluorescence-based spectroscopy is capable of detecting MW fields at strengths as low as 0.04 mV/cm, which is limited by the BBR background. More recently, Kaur et al. studied the influence of BBR on Rydberg-atom-based RF sensors by increasing the dephasing rate [58].

**Figure 7:**
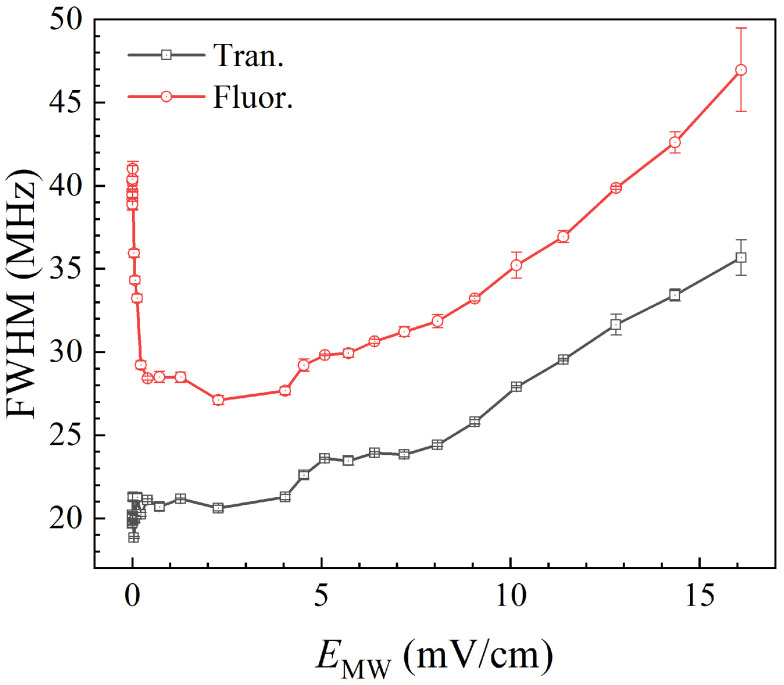
The FWHM variation in transmission and fluorescence spectra under medium MW fields from 0 to 18.1 mV/cm. The black hollow square represents the transmission readout, the red hollow circle represents the fluorescence readout, and the error bar is given by the short line.

Figure 7 shows experimental results of the full width at half maximum (FWHM) variation of fluorescence and transmission spectra with MW field strength from 0 to 18.1 mV/cm (corresponding to an MW source power set from −135 to −2 dBm). The black hollow square represents the transmission readout, the red hollow circle represents the fluorescence readout, and the error bar is given by the short line. In this range, as the MW field strength increases, the FWHM of the transmission spectrum remains constant first, then starts increasing after 4.0 mV/cm until splitting at 18.1 mV/cm, while the FWHM of the fluorescence spectrum follows the same trend. Under the medium MW field (>4.0 mV/cm, <18.1 mV/cm), the increase in the MW field strength causes small splits in the Rydberg energy levels and gradually rises, resulting in spectral broadening, until AT splitting exceeds the EIT linewidth. Therefore, in this range, the FWHM of the fluorescence spectral peak can be used to measure MW fields. Significantly, under an MW field ranging from 0 to 4.0 mV/cm, even though the FWHM of the transmission spectrum remains unchanged, the FWHM of the fluorescence spectrum first decreases and then stabilizes, with the turning point occurring precisely at an MW strength of 0.04 mV/cm, where the same field strength as the fluorescence peak amplitude begins to enhance, as shown in Figure 6. These observations confirm the influence of BBR-driven population transfer and enhanced dephasing, which has been recently reported by Kaur et al. in reference [58]: when the coherent MW field is comparable to or weaker than the BBR background, the EIT transmission is largely unchanged while incoherent BBR can produce an additional fluorescence peak; as the coherent MW field becomes larger than the effective BBR background (≈0.04 mV/cm in our data), coherent transfer likely becomes more significant, which can increase the fluorescence amplitude and reduce its FWHM before Autler–Townes broadening appears.

**Figure 8:**
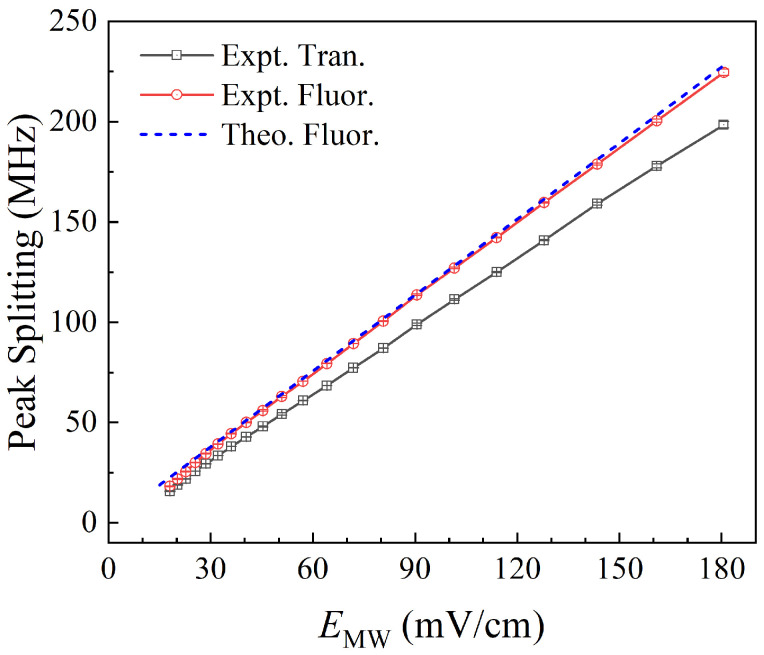
The AT splitting interval variation in transmission and fluorescence spectra under strong MW fields from 18.1 to 180.6 mV/cm. The black hollow square represents the transmission readout, the red hollow circle represents the fluorescence readout, the error bar is given by the short line, and the blue dashed line represents the theoretical result.

Figure 8 shows experimental results of the AT splitting interval of fluorescence and transmission spectra with MW field strengths from 18.1 to 180.6 mV/cm (corresponding to an MW source power set from −2 to 18 dBm). The black hollow square represents the transmission readout, the red hollow circle represents the fluorescence readout, the error bar is given by the short line, and the blue dashed line represents the theoretical result. In this range, as the MW field strength increases, both transmission and fluorescence spectra exhibit typical AT splitting. Under a strong MW field (>18.1 mV/cm), the increase in MW field strength causes a gradual enlargement of Rydberg level splitting, resulting in a greater spectral splitting distance. Therefore, in this range, the AT splitting interval of the fluorescence spectral peak can be used to measure MW fields.

**Figure 9:**
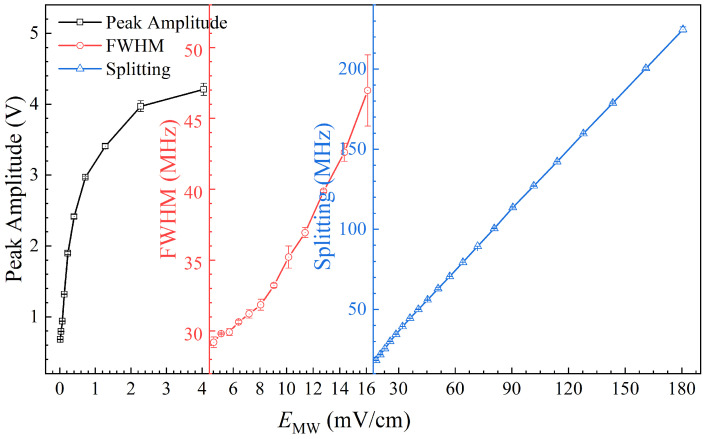
Wide-dynamic-range characterization by fluorescence detection. The horizontal axis of different regions adopts different scales, and the vertical axis divides different regions. The black hollow square represents the weak-field (<4.0 mV/cm) measurement by peak amplitude, the red hollow circle represents the medium-field (>4.0 mV/cm, <18.1 mV/cm) measurement by FWHM, the blue hollow triangle represents the strong-field (>18.1 mV/cm) measurement by AT splitting, and the error bar is given by the short line.

The experimental results indicate that the fluorescence detection scheme based on Rydberg atoms has the ability to measure MW field strength over a wide dynamic range, as shown in Figure 9. The horizontal axis of different regions adopts different scales, and the vertical axis divides different regions. The black hollow square represents the weak-field (>0.04 mV/cm, <4.0 mV/cm) measurement by the peak amplitude, the red hollow circle represents the medium-field (>4.0 mV/cm, <18.1 mV/cm) measurement by FWHM, the blue hollow triangle represents the strong-field (>18.1 mV/cm) measurement by AT splitting, and the error bar is given by the short line. This can achieve a wide-dynamic-range measurement of MW field strength, especially in weak MW fields, which cannot be achieved by traditional probe light transmission spectra. Fluorescence detection fully utilizes the advantages of weak fluorescence signal readouts to achieve MW weak-field measurements, which are limited by BBR backgrounds.

From the results in Figure 8, it can be seen that the EIT-AT splitting interval in the fluorescence spectra is larger than that in the transmission spectra. This phenomenon may be attributed to the fluorescence signal originating from a spatially non-uniform weighted summation, as the collection solid angle results in stronger signal detection from the central region compared to the peripheral areas, while the transmission spectrum comes from the integration along the entire light path. If the distribution of the MW field is non-uniform in the vapor cell, the fluorescence spectrum predominantly reflects contributions of MWs in the center regions of the cell. The transmission spectrum reflects the average result along the light propagation. Therefore, even when the antenna and the vapor cell satisfy the far-field condition, it remains necessary to investigate the distortion of the transmitted and reflected MW field by the wall of the vapor cell. In the following paragraph, the unique advantages of fluorescence detection will be used to measure the MW field distribution along the laser beam path within the vapor cell.

**Figure 10:**
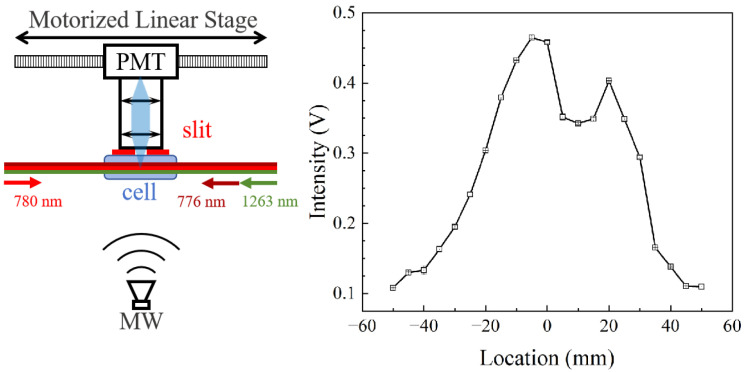
The experimental setup and result for spatial resolution using slits. The cell is 75 mm long. The 5 mm wide slit is positioned perpendicular to the laser propagation close to the vapor cell. It provides a spatial resolution of 6.7 mm, together with the coaxial fluorescence collection system. Other details are the same as those in Figure 3. The experimental setup is shown on the left, and the result is shown on the right.

The experimental setup and result for spatial resolution using slits are shown in Figure 10. We use a 75 mm long cell instead to obtain a longer scanning range. A slit with a 5 mm width is positioned perpendicular to the laser propagation close to the vapor cell, which can provide a spatial resolution of 6.7 mm, estimated by a simple simulation. The coaxial fluorescence collection system is put on a motorized linear stage to move along the laser propagation. Other details are the same as those in Figure 3. The micromave power is set at −25 dBm, where the peak amplitude of the fluorescence spectrum can be used to characterize the Rydberg excitation profile and MW fields. As the PMT + slit system moves from the left outer side of the vapor cell and then leaves from the other side, the fluorescence signal shows an initial increase, then remains unchanged, and, finally, decreases, as shown in Figure 10. The fluorescence intensity decreases with distance from the cell center, suggesting that the MW field strength distribution may be affected by the cell wall. Although the MW field was not fully resolved, this spatial dependence demonstrates the capability for spatially resolved fluorescence detection using the PMT + slit configuration. In the future, we will attempt to use a single-photon-sensitive CCD for direct imaging to achieve spatially resolved fluorescence MW measurements.

## 5. Conclusions

We theoretically and experimentally investigated, in detail, the fluorescence and transmission readouts of radio-frequency electrometry based on three-photon-excited Rydberg atoms. We developed theoretical models for the fluorescence and transmission readout processes of a three-photon-excited Rydberg-atom electrometer and performed a qualitative comparison of fluorescence versus probe transmission readout. Theoretical calculations revealed that while both fluorescence and probe transmission readouts can employ Autler–Townes splitting to measure strong RF fields, probe transmission readouts become ineffective in the weak-field regime, whereas fluorescence readouts remain sensitive to the RF field strength. Experimentally, we characterize the fluorescence response across a wide dynamic range of RF fields: from the weak-field regime (exhibiting a positive correlation of fluorescence peak amplitude under RF field strengths from 0.04 to 4.0 mV/cm, lower limited by BBR), through the medium-field regime (where the fluorescence spectral linewidth scales proportionally with RF field strengths from 4.0 to 18.1 mV/cm), to the strong-field regime (where the AT interval is linearly correlated with the RF field strength over 18.1 mV/cm). The achieved detection sensitivity for RF fields can reach levels as low as the background intensity of BBR. However, this also imposes stringent requirements on the stability of laser intensity. Employing lasers with higher stability is essential for achieving high-resolution RF field measurements via fluorescence detection. Furthermore, by adding a 5 mm narrow slit in front of the fluorescence collection system in close proximity to the vapor cell and scanning the collection system transversely across the excitation volume, we exploit fluorescence’s inherent spatial information to directly map the Rydberg excitation profile and local RF field strength. This overcomes the transmission readout’s inherent limitation of providing only path-integrated signals along the probe beam, even with imaging. Our results establish fluorescence readouts as a superior technique for three-photon Rydberg electrometry, offering enhanced wide-range RF field sensing and direct spatial field mapping.

## Data Availability

The original contributions presented in this study are included in the article. Further inquiries can be directed to the corresponding authors.

## Abbreviations

The following abbreviations are used in this manuscript:

RF	Radio frequency
SI	International System of Units
EIT	Electromagnetically induced transparency
Rb	Rubidium
AT	Autler–Townes
LIF	Laser-induced fluorescence
EIA	Electromagnetically induced absorption
PMT	Photomultiplier tube
MW	Microwave
AOM	Acousto-optic modulator
EOM	Electro-optic modulator
FWHM	Full width at half maximum
BBR	Blackbody radiation

## References

[1] Kobayashi T., Takamizawa A., Akamatsu D., Kawasaki A., Nishiyama A., Hosaka K., Hisai Y., Wada M., Inaba H., Tanabe T. (2022). Search for Ultralight Dark Matter from Long-Term Frequency Comparisons of Optical and Microwave Atomic Clocks. Phys. Rev. Lett..

[2] Hanneke D., Fogwell S., Gabrielse G. (2008). New Measurement of the Electron Magnetic Moment and the Fine Structure Constant. Phys. Rev. Lett..

[3] Bouchendira R., Cladé P., Guellati-Khélifa S., Nez F., Biraben F. (2011). New Determination of the Fine Structure Constant and Test of the Quantum Electrodynamics. Phys. Rev. Lett..

[4] Degen C.L., Reinhard F., Cappellaro P. (2017). Quantum Sensing. Rev. Mod. Phys..

[5] Ludlow A.D. (2015). Optical Atomic Clocks. Rev. Mod. Phys..

[6] Marti G.E., Hutson R.B., Goban A., Campbell S.L., Poli N., Ye J. (2018). Imaging Optical Frequencies with 100 µHz Precision and 1.1 µm Resolution. Phys. Rev. Lett..

[7] Zhang C., Ooi T., Higgins J.S., Doyle J.F., von der Wense L., Beeks K., Leitner A., Kazakov G.A., Li P., Thirolf P.G. (2024). Frequency Ratio of the 229mTh Nuclear Isomeric Transition and the 87Sr Atomic Clock. Nature.

[8] Müller H., Chiow S.w., Herrmann S., Chu S., Chung K.Y. (2008). Atom-Interferometry Tests of the Isotropy of Post-Newtonian Gravity. Phys. Rev. Lett..

[9] Zhan M., Xie X. (2020). Precision Measurement Physics: Physics That Precision Matters. Natl. Sci. Rev..

[10] Peters A., Chung K.Y., Chu S. (2001). High-Precision Gravity Measurements Using Atom Interferometry. Metrologia.

[11] Saywell J.C., Carey M.S., Light P.S., Szigeti S.S., Milne A.R., Gill K.S., Goh M.L., Perunicic V.S., Wilson N.M., Macrae C.D. (2023). Enhancing the Sensitivity of Atom-Interferometric Inertial Sensors Using Robust Control. Nat. Commun..

[12] Snadden M.J., McGuirk J.M., Bouyer P., Haritos K.G., Kasevich M.A. (1998). Measurement of the Earth’s Gravity Gradient with an Atom Interferometer-Based Gravity Gradiometer. Phys. Rev. Lett..

[13] Dang H.B., Maloof A.C., Romalis M.V. (2010). Ultrahigh Sensitivity Magnetic Field and Magnetization Measurements with an Atomic Magnetometer. Appl. Phys. Lett..

[14] Merlet S., Bodart Q., Malossi N., Landragin A., Santos F.P.D., Gitlein O., Timmen L. (2010). Comparison between Two Mobile Absolute Gravimeters: Optical versus Atomic Interferometers. Metrologia.

[15] Sedlacek J.A., Schwettmann A., Kübler H., Löw R., Pfau T., Shaffer J.P. (2012). Microwave Electrometry with Rydberg Atoms in a Vapour Cell Using Bright Atomic Resonances. Nat. Phys..

[16] Jing M., Hu Y., Ma J., Zhang H., Zhang L., Xiao L., Jia S. (2020). Atomic Superheterodyne Receiver Based on Microwave-Dressed Rydberg Spectroscopy. Nat. Phys..

[17] Ding D.S., Liu Z.K., Shi B.S., Guo G.C., Mølmer K., Adams C.S. (2022). Enhanced Metrology at the Critical Point of a Many-Body Rydberg Atomic System. Nat. Phys..

[18] Fan H., Kumar S., Sedlacek J., Kübler H., Karimkashi S., Shaffer J.P. (2015). Atom Based RF Electric Field Sensing. J. Phys. B At. Mol. Opt. Phys..

[19] Simons M.T., Gordon J.A., Holloway C.L., Anderson D.A., Miller S.A., Raithel G. (2016). Using Frequency Detuning to Improve the Sensitivity of Electric Field Measurements via Electromagnetically Induced Transparency and Autler-Townes Splitting in Rydberg Atoms. Appl. Phys. Lett..

[20] Zhang L., Jia Y., Jing M., Guo L., Zhang H., Xiao L., Jia S. (2019). Detuning Radio-Frequency Electrometry Using Rydberg Atoms in a Room-Temperature Vapor Cell. Laser Phys..

[21] Jia F., Yu Y., Liu X., Zhang X., Zhang L., Wang F., Mei J., Zhang J., Xie F., Zhong Z. (2020). Dispersive Microwave Electrometry Using Zeeman Frequency Modulation Spectroscopy of Electromagnetically Induced Transparency in Rydberg Atoms. Appl. Opt..

[22] Shi Y., Li C., Ouyang K., Ren W., Li W., Cao M., Xue Z., Shi M. (2023). Tunable Frequency of a Microwave Mixed Receiver Based on Rydberg Atoms under the Zeeman Effect. Opt. Express.

[23] Li X., Cui Y., Hao J., Zhou F., Wang Y., Jia F., Zhang J., Xie F., Zhong Z. (2023). Magnetic-Field-Induced Splitting of Rydberg Electromagnetically Induced Transparency and Autler-Townes Spectra in ^87^Rb Vapor Cell. Opt. Express.

[24] Schlossberger N., Rotunno A.P., Artusio-Glimpse A.B., Prajapati N., Berweger S., Shylla D., Simons M.T., Holloway C.L. (2024). Zeeman-Resolved Autler-Townes Splitting in Rydberg Atoms with Tunable Resonances and a Single Transition Dipole Moment. Phys. Rev. A.

[25] Jia F.D., Liu X.B., Mei J., Yu Y.H., Zhang H.Y., Lin Z.Q., Dong H.Y., Zhang J., Xie F., Zhong Z.P. (2021). Span Shift and Extension of Quantum Microwave Electrometry with Rydberg Atoms Dressed by an Auxiliary Microwave Field. Phys. Rev. A.

[26] Simons M.T., Artusio-Glimpse A.B., Holloway C.L., Imhof E., Jefferts S.R., Wyllie R., Sawyer B.C., Walker T.G. (2021). Continuous Radio-Frequency Electric-Field Detection through Adjacent Rydberg Resonance Tuning. Phys. Rev. A.

[27] Yuan J., Jin T., Wang L., Xiao L., Jia S. (2022). Improvement of Microwave Electric Field Measurement Sensitivity via Dual-Microwave-Dressed Electromagnetically Induced Transparency in Rydberg Atoms. Laser Phys. Lett..

[28] Liu X.H., Liao K.Y., Zhang Z.X., Tu H.T., Bian W., Li Z.Q., Zheng S.Y., Li H.H., Huang W., Yan H. (2022). Continuous-Frequency Microwave Heterodyne Detection in an Atomic Vapor Cell. Phys. Rev. Appl..

[29] Cui Y., Jia F.D., Hao J.H., Wang Y.H., Zhou F., Liu X.B., Yu Y.H., Mei J., Bai J.H., Bao Y.Y. (2023). Extending Bandwidth Sensitivity of Rydberg-atom-based Microwave Electrometry Using an Auxiliary Microwave Field. Phys. Rev. A.

[30] Liu X., Jia F., Zhang H., Mei J., Yu Y., Liang W., Zhang J., Xie F., Zhong Z. (2021). Using Amplitude Modulation of the Microwave Field to Improve the Sensitivity of Rydberg-atom Based Microwave Electrometry. AIP Adv..

[31] Hao J.H., Jia F.D., Cui Y., Wang Y.H., Zhou F., Liu X.B., Zhang J., Xie F., Bai J.H., You J.Q. (2024). Microwave Electrometry with Rydberg Atoms in a Vapor Cell Using Microwave Amplitude Modulation. Chin. Phys. B.

[32] Gomes N.D., Pepino V.M., Borges B.H.V., Magalhães D.V., de Jesus Napolitano R., Torres M.A.L., Kondo J.D.M., Marcassa L.G. (2024). Rydberg Atom-Based Microwave Electrometry Using Polarization Spectroscopy. J. Phys. B At. Mol. Opt. Phys..

[33] Liao K.Y., Tu H.T., Yang S.Z., Chen C.J., Liu X.H., Liang J., Zhang X.D., Yan H., Zhu S.L. (2020). Microwave Electrometry via Electromagnetically Induced Absorption in Cold Rydberg Atoms. Phys. Rev. A.

[34] Zhou F., Jia F., Liu X., Yu Y., Mei J., Zhang J., Xie F., Zhong Z. (2023). Improving the Spectral Resolution and Measurement Range of Quantum Microwave Electrometry by Cold Rydberg Atoms. J. Phys. B At. Mol. Opt. Phys..

[35] Zhou F., Jia F.D., Liu X.B., Zhang J., Xie F., Zhong Z.P. (2023). Measurement of Microwave Electric Field Based on Electromagnetically Induced Transparency by Using Cold Rydberg Atoms. Acta Phys. Sin..

[36] Kumar S., Fan H., Kübler H., Jahangiri A.J., Shaffer J.P. (2017). Rydberg-Atom Based Radio-Frequency Electrometry Using Frequency Modulation Spectroscopy in Room Temperature Vapor Cells. Opt. Express.

[37] Gordon J.A., Simons M.T., Haddab A.H., Holloway C.L. (2019). Weak Electric-Field Detection with Sub-1 Hz Resolution at Radio Frequencies Using a Rydberg Atom-Based Mixer. AIP Adv..

[38] Cai M., You S., Zhang S., Xu Z., Liu H. (2023). Sensitivity Extension of Atom-Based Amplitude-Modulation Microwave Electrometry via High Rydberg States. Appl. Phys. Lett..

[39] Holloway C.L., Prajapati N., Artusio-Glimpse A.B., Berweger S., Simons M.T., Kasahara Y., Alù A., Ziolkowski R.W. (2022). Rydberg Atom-Based Field Sensing Enhancement Using a Split-Ring Resonator. Appl. Phys. Lett..

[40] Liu B., Zhang L.H., Wang Q.F., Ma Y., Han T.Y., Liu Z.K., Zhang Z.Y., Shao S.Y., Zhang J., Li Q. (2025). Cavity-Enhanced Rydberg Atom Microwave Receiver. Chin. Phys. Lett..

[41] Wang Q., Liang Y., Wang Z., Guan S., Yang P., Zhang P., Li G., Zhang T. (2025). High-Precision Measurement of Microwave Electric Field by Cavity-Enhanced Critical Behavior in a Many-Body Rydberg Atomic System. Sci. China Phys. Mech. Astron..

[42] Yang W., Jing M., Zhang H., Zhang L., Xiao L., Jia S. (2023). Enhancing the Sensitivity of Atom-Based Microwave-Field Electrometry Using a Mach-Zehnder Interferometer. Phys. Rev. Appl..

[43] Holloway C.L., Gordon J.A., Schwarzkopf A., Anderson D.A., Miller S.A., Thaicharoen N., Raithel G. (2014). Sub-Wavelength Imaging and Field Mapping via Electromagnetically Induced Transparency and Autler-Townes Splitting in Rydberg Atoms. Appl. Phys. Lett..

[44] Fan H.Q., Kumar S., Daschner R., Kübler H., Shaffer J.P. (2014). Subwavelength Microwave Electric-Field Imaging Using Rydberg Atoms inside Atomic Vapor Cells. Opt. Lett..

[45] Walker D.A. (1987). A Fluorescence Technique for Measurement of Concentration in Mixing Liquids. J. Phys. E Sci. Instrum..

[46] Prajapati N., Berweger S., Rotunno A.P., Artusio-Glimpse A.B., Schlossberger N., Shylla D., Watterson W.J., Simons M.T., LaMantia D., Norrgard E.B. (2024). Investigation of Fluorescence versus Transmission Readout for Three-Photon Rydberg Excitation Used in Electrometry. AVS Quantum Sci..

[47] Holloway C.L., Simons M.T., Gordon J.A., Dienstfrey A., Anderson D.A., Raithel G. (2017). Electric Field Metrology for SI Traceability: Systematic Measurement Uncertainties in Electromagnetically Induced Transparency in Atomic Vapor. J. Appl. Phys..

[48] Miller B.N., Meyer D.H., Virtanen T., O’Brien C.M., Cox K.C. (2024). RydIQule: A Graph-Based Paradigm for Modeling Rydberg and Atomic Sensors. Comput. Phys. Commun..

[49] Šibalić N., Pritchard J.D., Adams C.S., Weatherill K.J. (2017). ARC: An Open-Source Library for Calculating Properties of Alkali Rydberg Atoms. Comput. Phys. Commun..

[50] Gordon J.A., Holloway C.L., Schwarzkopf A., Anderson D.A., Miller S., Thaicharoen N., Raithel G. (2014). Millimeter Wave Detection via Autler-Townes Splitting in Rubidium Rydberg Atoms. Appl. Phys. Lett..

[51] Gordon E.I. (1966). A Review of Acoustooptical Deflection and Modulation Devices. Appl. Opt..

[52] van Wijngaarden W.A., Li J. (1997). Calibration of Laser Frequency Scan with an Electro-Optic Modulator. Appl. Opt..

[53] Kessler T., Hagemann C., Grebing C., Legero T., Sterr U., Riehle F., Martin M.J., Chen L., Ye J. (2012). A Sub-40-mHz-linewidth Laser Based on a Silicon Single-Crystal Optical Cavity. Nat. Photon.

[54] Udem T., Holzwarth R., Hänsch T.W. (2002). Optical Frequency Metrology. Nature.

[55] Rosenband T., Hume D.B., Schmidt P.O., Chou C.W., Brusch A., Lorini L., Oskay W.H., Drullinger R.E., Fortier T.M., Stalnaker J.E. (2008). Frequency Ratio of Al+ and Hg+ Single-Ion Optical Clocks—Metrology at the 17th Decimal Place. Science.

[56] Morris M.B., McIlrath T.J., Snyder J.J. (1984). Fizeau Wavemeter for Pulsed Laser Wavelength Measurement. Appl. Opt..

[57] Robertson E.J., Šibalić N., Potvliege R.M., Jones M.P.A. (2021). ARC 3.0: An Expanded Python Toolbox for Atomic Physics Calculations. Comput. Phys. Commun..

[58] Kaur C., Shen P., Booth D., Todd A., Shaffer J.P. (2025). The Impact of Thermal Fields on Rydberg Atom Radio Frequency Sensors. arXiv.

